# Nutritional State, Immunological and Biochemical Parameters, and Mortality in the ICU: An Analytical Study

**DOI:** 10.3390/jcm12134177

**Published:** 2023-06-21

**Authors:** Blanca Cecilia Díaz Chavarro, Manuel Romero-Saldaña, Jorge Karim Assis Reveiz, Guillermo Molina-Recio

**Affiliations:** 1 Nursing Program, School of Health, Institute of Biomedical Research (IIB), Universidad Santiago de Cali, Santiago de Cali 760001, Colombia; blanca.diaz00@usc.edu.co; 2 Doctoral Program in Biosciences and Agricultural and Food Sciences, University of Córdoba, 14014 Cordoba, Spain; 3 Nursing, Pharmacology and Physiotherapy Department, University of Córdoba, 14004 Cordoba, Spain; gmrsurf75@gmail.com; 4 Lifestyles, Innovation and Health (GA—16), Maimonides Biomedical Research Institute of Córdoba (IMIBIC), 14014 Cordoba, Spain; 5 Department of Research and Education, Clínica de Occidente SA, Santiago de Cali 760001, Colombia; ophthalmology2013@gmail.com

**Keywords:** malnutrition, obesity, leukocytosis, lymphocyte, critical care

## Abstract

Intensive care unit (ICU) hospitalization involves critically ill patients with multiple diseases and possible complications, including malnutrition, which increases hospital stay and mortality. Therefore, identifying the patient’s prior nutritional state, following up during hospitalization, and implementing early intervention positively affect patient’s vital situations at discharge. The objective of this study is to determine the nutritional state of patients admitted to an ICU in Cali (Colombia) in 2019 and its association with immunological and biochemical parameters and mortality observed during hospitalization. This was an observational, analytical, and retrospective study of patients admitted to an ICU in a clinic in Cali (Colombia) from 1 January to 31 March 2019. The association between their nutritional state and outcome variables such as hospital stay, immunological and biochemical function, and mortality was analyzed. Logistic regression was used to predict patients’ vital status at hospital discharge. In terms of the nutritional level, low weight was observed in 17.5% patients, and overweight/obesity was observed in 53.5% of the population. Nutritional state was associated with leukocytosis. The patients with lymphocytosis had longer hospital stays than those with normal lymphocyte ranges. Age, blood leukocytes, and creatinine and potassium levels increased the risk of mortality. Lymphocyte values have been used as predictors of severity and hospitalization time. The scientific literature has also evidenced a higher leukocyte count in people with obesity, and such leukocytosis is associated with the risk of mortality. The results of blood and laboratory tests determining kidney function and blood electrolytes allow for the prediction of mortality risk in critically ill patients.

## 1. Introduction

At the epidemiological level, disease-related malnutrition is one of the major public health concerns worldwide, [1] affecting 30–50% of ICU patients [2]. This problem is linked to the hypercatabolic and hypermetabolic state of critically ill patients [3], which is associated with those who require advanced support for the maintenance of hemodynamic variables [4], those with reduced mobility, and those with reduced food intake who are at increased risk of malnutrition, which can be exacerbated in older adults [5,6,7].

This condition increases patient morbidity, including complications such as infections, wound dehiscence, and delayed fracture consolidation, thereby extending hospital stay and increasing readmission and mortality rates [8]. For instance, in the study conducted by the American Society for Parenteral and Enteral Nutrition (ASPEN) in a Pennsylvania hospital, it was found that the average hospitalization time was 14.99 days in malnourished patients and 11.85 in non-malnourished patients. In addition, readmissions were higher in malnourished patients (40% vs. 23%), as were mortality (8% vs. 5%) [9].

In Latin American hospitals, malnutrition has been found in up to 74.1% of critically ill patients using the Subjective Global Assessment (SGA), and 39.2% were identified as being at risk of poor clinical outcomes according to the NUTRIC score [10]. Patients admitted to intensive care units (ICUs) should be managed using a comprehensive care [11] and holistic approach, which includes assessing the patient’s nutritional state [4] and taking into account factors such as age, pathology, and clinical characteristics that affect malnutrition and increase the risk of mortality [12]. These variables constitute a population’s epidemiological profile, which is defined as the clinical status analyzed at a certain place and time [13]. Understanding the epidemiological variables and nutritional status of ICU patients, monitoring its variations throughout hospitalization, and implementing early and prompt intervention measures have a positive impact on the patient’s clinical outcome [10,14] and are essential for their management [15] because they improve the standardization of specialized health care processes [11].

The purpose of this study was to determine the nutritional state of patients admitted to an ICU in Cali (Colombia) in 2019 and its association with immunological and biochemical parameters and mortality observed during hospitalization.

## 2. Materials and Methods

### 2.1. Design, Population, and Sample

This was an observational, analytical, and retrospective study of patients admitted to the ICU of a clinic in Cali, Colombia, from 1 January to 31 March 2019. The ICU consisted of 5 modules with the following patient capacity: surgical module (8 patients), cardiovascular module (9 patients), coronary or intermediate module (6 patients), general module (11 patients), and neurovascular module (10 patients). The Strengthening the Reporting of Observational Studies in Epidemiology (STROBE) guidelines for observational studies were followed.

Participants were patients admitted to the ICU of a clinic in Cali, Colombia, between 1 January and 31 March 2019. The data recorded in the systematized clinical history during the process of care within the service were reviewed; this review was carried out during the year 2022, after approval by the ethics and research committee of the institution.

The overall population consisted of 336 patients admitted during that period. In order to obtain an 80% disease-related malnutrition risk for patients receiving routine health care [16,17,18] a 6% accuracy, and a 95% safety, the sample size was 114 patients.

### 2.2. Elegibity Criteria

The following inclusion criteria were applied: patients admitted to the ICU from January to March 2019 and aged ≥18 years.

**Figure 1:**
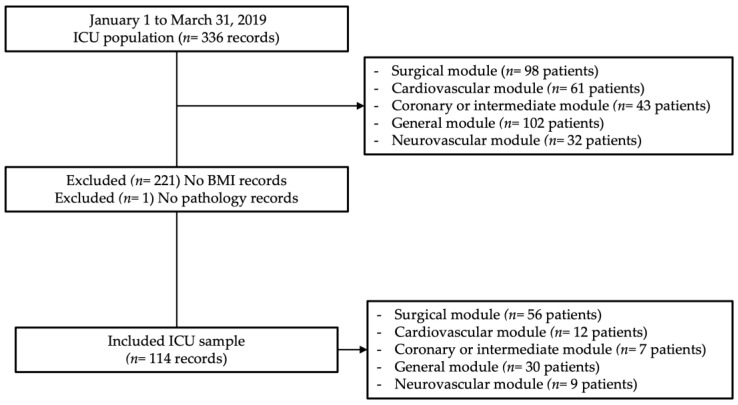
Sample selection flow diagram. Abbreviations: n, number; ICU, Intensive Care Unit; BMI, Body Mass Index.

Patients whose nutritional state could not be determined or whose sociodemographic data was incomplete were excluded (Figure 1).

### 2.3. Variables and Measurement

The following outcome variables were identified: (i) ICU stay in days, (ii) vital situation (deceased or alive), and (iii) immunological function with blood leukocyte test (normal range = 4.5–11 × 10^3^/uL, leukocytosis > 11 × 10^3^/uL, and leukopenia < 4.5 × 10^3^/uL) and blood lymphocytes (normal range = 17–45%, lymphocytosis > 45%, and lymphopenia < 17%).

Explanatory variables were as follows: sex, age (years), life course classification according to Resolution no. 3280 of Colombia, 2018 [19] (which identifies young subjects as being 18–28 years of age, adults as being 29–59 years of age, and the elderly as being >60 years of age), medical diagnosis upon admission based on the International Classification of Diseases (ICD-10), weight (kg), size (cm), body mass index—BMI—(<18.5 kg/m^2^ underweight, 18.5–24.9 kg/m^2^ normal weight, 25.0–29.9 kg/m^2^ overweight and ≥30.0 kg/m^2^ obese), clinical impression of BMI (low weight, normal weight, and overweight or obesity), and serum creatinine (mg/dL) and potassium (mEq/L) levels.

The data concerning the nutritional status of the patients were limited to the BMI as the anthropometric parameter used in the care of the patients treated in this ICU, accompanied by the clinical impression of the BMI, which was determined by the nutrition and dietetics staff of the institution. Immunological and biochemical data were added to complement this information and allow a more complete analysis of the patient’s state of health.

### 2.4. Ethical Aspects

This study has been approved by the Research Ethics and Bioethics Scientific Committee and the Technical–Scientific Committee of the Hospital (record IYECDO—1358). Moreover, the study followed the Helsinki Declaration and the ethical principles for research with human beings, ensuring their health. Each participant or their legal representative signed the informed consent prior to data collection.

### 2.5. Statistical Analysis

IBM SPSS version 28.0 statistical software was used. Quantitative variables were represented by their arithmetic means and standard deviations, while qualitative variables were summarized by their absolute and relative frequencies. Pearson’s chi-square test was used to compare categorical variables, applying Fisher’s exact test when necessary. To compare two or more arithmetic means, Student’s t test or single factor analysis of variance was used depending on the case. Subgroup analyses were applied to interpret the interaction between nutritional state and outcome variables, such as hospital stay, immunological function, and vital situation. Statistical significance was established at a *p*-value < 0.05. Kolmogorov–Smirnov’s normality test with Lilliefors correction was applied for continuous quantitative variables. Multiple logistic regression was conducted by determining adjusted OR values, the model’s goodness of fit through deviance, Cox and Snell and Nagelkerke’s coefficients of determination, and the Hosmer–Lemeshow statistic. Moreover, the model’s diagnostic accuracy and discriminating capacity were determined by estimating the area under the ROC curve and validity and safety indicators such as sensitivity, specificity, predictive values, and validity index.

## 3. Results

### 3.1. Descriptive Profile of ICU Patients

Of the 114 participants, 66 (57.9%) were women. The global mean age was 62.38 years (SD = 17.63; 95% CI: 59.1–65.7). Oral feeding was provided to 68.4% of patients on admission, and only one patient required mixed nutritional support.

In terms of the nutritional level, low weight was observed in 20 patients (17.5%) and overweight/obesity was observed in 53.5% of the population. In terms of immunological function, an average of 18.33 × 10^3^/uL leukocytes were observed (SD = 36.10; 95% CI: 11.6–25). The average lymphocyte level was 15.16 (SD = 11.49; 95% CI: 13–17.3), with a higher trend toward lymphopenia (64.9%). Biochemical parameters yielded an average creatinine level of 1.61 (SD = 2.70; 95% CI: 1.1–2.1) and a mean potassium level of 4.10 (SD = 0.71; 95% CI: 4–4.2).

The highest proportion of participants consisted of subjects aged ≥60 years (58.8%), and the mean hospitalization day was 23.96 (SD = 23.85; 95% CI: 19.5–28.4). In the hospitalized population, the prevalence of circulatory and respiratory diseases was 31.6%, followed by 29.8% for neoplasms. The mortality rate was 37.7% (Table 1).

### 3.2. Factors Associated with Hospital Stay

Among the variables identified in ICU patients, an association was found between diseases at the time of admission and hospital stay (*p* = 0.042), with patients with neoplastic diseases having a longer average stay (33.26; SD = 29.22; 95% CI: 23.1–43.5) and patients with circulatory and respiratory diseases having a shorter average stay (17.81; SD = 19.17; 95% CI: 11.3–24.3).

A comparison of hospital stay means among patients with different lymphocyte ranges yielded a significant difference (*p* = 0.04), with patients with lymphocytosis having longer hospital stays (47.33, SD = 49.31; 95% CI: 4.0–101) than those with normal lymphocyte ranges (17.41, SD = 17.72; 95% CI: 11.5–23.3) (Table 2).

### 3.3. Factors Associated with Immunological Function

The mean age of patients with leukocytosis was lower (58.44 years; SD = 19.18; 95% CI: 53.2–63.7). Moreover, this immunological alteration was more common in subjects with infectious (56.3%) and neoplastic (55.9%) diseases.

A significant association was observed between nutritional state and immunological function (*p* = 0.018), with ill patients with normal weight and overweight/obesity showing a higher tendency toward leukocytosis (Table 3).

### 3.4. Factors Associated with Vital Situation

The group of patients who died had a higher mean age (66.26 years, SD = 17.08; 95% CI: 61–71.5). In terms of life course, 40.3% of patients who died were elderly, and mortality was higher in patients with neoplastic diseases (44.1%) and low weight (55%) (Table 4).

### 3.5. Multivariate Analysis of ICU Mortality

The variables with statistical significance in the raw analysis were used to adjust logistic regression. Table 5 shows the adjusted model that predicts mortality. For every year of age and leukocyte unit, mortality risk increases 1.02 times, whereas it increases 2.9 times for every 1 mEq/L increase in potassium level.

This model predicted a 46.5% mortality rate among ICU patients versus the 53.5% of the mortality rate actually observed, and it has a 91.5% negative predictive value, which is more accurate when the patient is discharged alive. It has a sensitivity of 76.9% and a specificity of 73.9%, with a capacity of 72.3% to distinguish the vital situations of patients leaving the ICU.

## 4. Discussion

The nutritional state of 114 patients admitted to an ICU in Cali (Colombia) in 2019 and its association with immunological and biochemical parameters and mortality was observed during hospitalization.

### 4.1. Prevalence of Diseases upon Admission and Mortality in the ICU

When studying the morbidity and mortality of ICU patients, a significant prevalence of circulatory and respiratory diseases was observed, followed by neoplastic diseases; this is consistent with the study by Nachvak et al. (2018) in Iran [20], who first described diseases such as kidney failure with hypertension, chronic obstructive pulmonary disease with thoracic surgery, and unspecified pulmonary disease (30.8%). The mortality rate observed in our study is consistent with the one reported globally among ICUs, between 17.2% in patients without healthcare-associated infections (HAI) and those with 1 HAI from 30.15% to 48.21% [21].

### 4.2. Diseases upon Admission and Lymphocytosis during ICU Stay

In terms of factors associated with hospital stay, patients with oncological diseases had the longest mean ICU stay, which is consistent with what was reported by Hernández-Tejedor et al. (2015) in a multicenter study conducted in Spain, in which a history of oncological disease or immunosuppressive therapy were identified as factors affecting complications and increasing hospital stay [22].

Studies on the relationship between lymphocyte levels and hospital stay are scarce and have mainly focused on neutrophilia and lymphopenia as inflammatory responses, which increase the neutrophil–lymphocyte ratio (NLR), implying a differential role as a mortality predictor [23]. This ratio has been mainly used in patients with renal pathologies, abdominal surgeries, cardiovascular surgeries, trauma patients, and infectious pathologies [24,25,26]. In this case, lymphocyte count, which was classified into different ranges based on increased or decreased blood levels, showed an association with ICU average stay, suggesting a longer stay in patients with lymphocytosis regardless of the disease at the time of admission or neutrophil levels and NLR.

### 4.3. Nutritional State, Hospital Stay, and Immunological Function

No significant association was found when the nutritional state and ICU stay were studied, which is consistent with the study conducted by Lew et al. (2017) in a hospital in Singapore, in which no significant differences were observed in the ICU stay of patients with malnutrition [27]. However, Gong et al. (2010) conducted a study in Boston and reported a longer average ICU stay in the group of obese and severely obese patients [28].

On the contrary, the association between nutritional state and leukocyte immune function was significant, as also evidenced in the scientific literature with a higher leukocyte count among obese subjects [29]. Herishanu et al. (2006) conducted a study in Israel and reported that for every 1 kg/m^2^ increase in BMI, the risk of leukocytosis increased by 11% [30].

### 4.4. Nutritional State and Leukocyte Count in Mortality

As an anthropometric measure, BMI allows patients’ nutritional status to be established and classified [31,32,33]. According to the literature, its association with patient mortality and with the score obtained during the application of the NRS-2002 nutritional risk scale has been identified [34].

Nutritional state was not associated with patients’ vital situations when discharged from the ICU in Cali. This association was heterogeneous across the studies reviewed, and no association between obesity and mortality was observed in three publications [35,36,37]. However, a decrease in mortality was also reported in critically ill with overweight and obesity [38], both in the short and long term [39]. Tocalini et al. (2020) conducted seven systematic reviews and found varying results and statistical heterogeneity among studies [40].

When studying the association between leukocytosis and mortality, it was found that, similar to our results, a study conducted by Abete et al. (2019) in the Netherlands reported that white blood count could be used as a prediagnostic factor associated with the risk of all-cause mortality in critically ill patients [41].

### 4.5. Multivariate Analysis of ICU Mortality

Mortality has been a constant concern given the state of patients admitted to the ICU as well as the multiple diseases they may present, so several tools have been developed to measure severity and predict the likelihood of mortality. Some of the widely used models include Acute Physiology and Chronic Health Evaluation (APACHE) versions I, II, III, and IV and the Simplified Acute Physiology Score (SAPS) scale versions I, II, and III. APACHE II can adequately classify 80.9% of cases with 93% sensitivity. SAPS III can adequately classify 95% of patients whose deaths were predicted. However, both models had low specificity in the assessment of critically ill patients (SAPS III: 36.8% and APACHE II: 41.7%) [42]. When comparing the use of these models in ICU coronary care units, a sensitivity of 80% and a specificity of 91.8% were found when applying SAPS II. In comparison, the APACHE II model achieved a sensitivity of 75.9% and a specificity of 87.4% for predicting mortality. However, in this case, they are limited to prediction in patients treated for cardiovascular emergencies [43].

The ICU mortality-predicting model developed in this study includes data on age, leukocyte rate, and blood creatinine and potassium levels, all of which can be easily collected using basic laboratory tests to assess patients’ health status upon admission and during follow-up. The model had 76.9% and 73.9% sensitivity and specificity, respectively, and reached a 74.6% validity index (accurate classification) and a 91.5% negative predictive value, so it is better at predicting the “alive” vital situation than the abovementioned tools. It can be applied to generate the prognosis of all critically ill patients regardless of their admitting pathology.

### 4.6. Study Limitations and Strengths

A study limitation was the amount of missing data about patients’ nutritional state during ICU stay, which reduced the sample size. In addition, this status is based on BMI as an anthropometric indicator, which may limit the accuracy of the nutritional diagnosis. It makes it necessary to take the results with caution.

Including patients from all ICU units was a study strength because it resulted in a population with multiple admission causes, representing the variability of critically ill patients and their outcomes throughout the health–disease process.

## 5. Conclusions

The nutritional state affects the occurrence of leukocytosis, and this immunological function is associated with the critical patient’s vital situation when leaving the ICU, because the higher the leukocytosis, the higher the mortality risk. Lymphocytes, which are white blood cells, have been associated with hospital stays. When these results are interpreted based on blood and biochemical tests assessing kidney function and electrolyte levels, patients’ mortality risk or survival can be significantly predicted.

## Figures and Tables

**Table 1:** Descriptive profile of intensive care unit patients.

Variables	Total $\bar{x}$ (±SD) (*n*)%	Women (*n* = 66) $\bar{x}$ (±SD) (*n*)%	Men (*n* = 48) $\bar{x}$ (±SD) (*n*)%	*p*
Age	62.38 (±17.63)	61.52 (±17.41)	63.56 (±18.04)	0.543
Type of nutritional support				
Oral feeding	(78) 68.4%	(48) 72.7%	(30) 62.5%	0.296
Enteral nutritional support	(31) 27.2%	(17) 25.8%	(14) 29.2%	
Parenteral nutritional support	(4) 3.5%	(1) 1.5%	(3) 6.3%	
Mixed nutritional support	(1) 0.9%	(0)	(1) 2.1%	
Nutritional state				
Low weight	(20) 17.5%	(10) 15.2%	(10) 20.8%	0.725
Normal weight	(33) 29%	(20) 30.3%	(13) 27.1%	
Overweight/obesity	(61) 53.5%	(36) 54.5%	(25) 52.1%	
Immunological and biochemical functions				
Leukocytes (×10^3^/uL)	18.33 (±36.10)	19.40 (±42.67)	16.85 (±24.75)	0.352
Normal-range leukocytes	(46) 40.4%	(27) 40.9%	(19) 39.6%	0.484
Leukocytosis	(54) 47.4%	(29) 43.9%	25) 52.1%	
Leukopenia	(14) 12.3%	(10) 15.2%	(4) 8.3%	
Lymphocytes (%)	15.16 (±11.49)	15.28 (±10.43)	15.01 (±12.91)	0.635
Normal-range lymphocytes	(37) 32.5%	(24) 36.4%	(13) 27.1%	0.434
Lymphocytosis	(3) 2.6%	(1) 1.5%	(2) 4.2%	
Lymphopenia	(74) 64.9%	(41) 62.1%	(33) 68.8%	
Creatinine (mg/dL)	1.61 (±2.70)	1.60 (±2.95)	1.63 (±2.35)	0.948
Potassium (mEq/L)	4.10 (±0.71)	4.08 (±0.77)	4.13 (±0.63)	0.685
Disease upon admission				
Circulatory and respiratory diseases	(36) 31.6%	(20) 55.6%	(16) 44.4%	0.381
Neoplastic diseases	(34) 29.8%	(17) 50%	(17) 50%	
Other diseases	(28) 24.6%	(20) 71.4%	(8) 28.6%	
Infectious diseases	(16) 14%	(9) 56.3%	(7) 43.8%	
Vital situation				
Deceased	(43) 37.7%	(25) 58.1%	(18) 41.9%	0.967
Alive	(71) 62.3%	(41) 57.8%	(30) 42.3%	
Hospital stay (days)	23.96 (±23.85)	24.08 (±23.50)	23.81 (±24.56)	0.954

Abbreviations: *n*, number; $\bar{x}$, average; SD, Standard Deviation.

**Table 2:** Factors associated with hospital stay.

Variables	Hospital Stay (Days) $\bar{x}$ (±SD)	*p*
Age	Rho 0.016	0.868
Disease upon admission		
Circulatory and respiratory diseases	17.81 (±19.17)	0.042
Neoplastic diseases	33.26 (±29.22)	
Other diseases	22.96 (±22.89)	
Infectious diseases	19.81 (±17.25)	
Nutritional state		
Low weight	20.80 (±21.74)	0.601
Normal weight	27.24 (±22.66)	
Overweight/obesity	23.23 (±25.25)	
Immunological function		
$\bar{x}$ leukocytes (×10^3^/uL)	Rho −0.040	0.669
Normal-range leukocytes	18.89 (±18.29)	0.168
Leukocytosis	27.83 (±26.63)	
Leukopenia	25.71 (±27.14)	
$\bar{x}$ lymphocytes (%)	Rho −0.063	0.502
Normal-range lymphocytes	17.41 (±17.72)	0.040
Lymphocytosis	47.33 (±49.31)	
Lymphopenia	26.30 (±24.68)	

Abbreviations: *n*, number; $\bar{x}$, average; SD, Standard Deviation.

**Table 3:** Factors associated with immunological function.

Variables	Normal-Range Leukocytes $\bar{x}$ (±SD) (*n*)%	Leukocytosis $\bar{x}$ (±SD) (*n*)*%*	Leukopenia $\bar{x}$ (±SD) (*n*)%	*p*
Age	66.33 (±15.46)	58.44 (±19.18)	64.57 (±15.77)	0.073
Life course classification				
Youth	(1) 11.1%	(8) 88.9%	(0) 0%	0.121
Adults	(15) 39.5%	(17) 44.7%	(6) 15.8%	
Elderly	(30) 44.8%	(29) 43.3%	(8) 11.9%	
Disease upon admission				
Circulatory and respiratory diseases	(21) 58.3%	(11) 30.6%	(4) 11.1%	0.216
Neoplastic diseases	(11) 32.4%	(19) 55.9%	(4) 11.8%	
Other diseases	(10) 35.7%	(15) 53.6%	(3) 10.7%	
Infectious diseases	(4)25%	(9) 56.3%	(3) 18.8%	
Nutritional state				
Low weight	(6) 30%	(7) 35%	(7) 35%	0.018
Normal weight	(13) 39.4%	(17) 51.5%	(3) 9.1%	
Overweight/obesity	(27) 44.3%	(30) 49.2%	(4) 6.6%	

Abbreviations: *n*, number; $\bar{x}$, average; SD, Standard Deviation.

**Table 4:** Factors associated with vital situation.

Variables	Deceased $\bar{x}$ (±SD) (*n*)*%*	Alive $\bar{x}$ (±SD) (*n*)*%*	*p*
Age	66.26 (±17.08)	60.03 (±17.66)	0.067
Life course			
Youth	(2) 22.2%	(7) 77.8%	0.571
Adults	(14) 36.8%	(24) 63.2%	
Elderly	(27) 40.3%	(40) 59.7%	
Disease upon admission			
Circulatory and respiratory diseases	(12) 33.3%	(24) 66.7%	0.512
Neoplastic diseases	(15) 44.1%	(19) 55.9%	
Other diseases	(12) 42.9%	(16) 57.1%	
Infectious diseases	(4) 25%	(12) 75%	
Nutritional state			
Low weight	(11) 55%	(9) 45%	0.157
Normal weight	(13) 39.4%	(20) 60.6%	
Overweight/obesity	(19) 31.1%	(42) 68.9%	
Immunological function			
Leukocytes (×10^3^/uL)	27.82 (±56.83)	12.58 (±8.79)	0.028
Normal-range leukocytes	(12) 26.1%	(34) 73.9%	0.066
Leukocytosis	(23) 42.6%	(31) 57.4%	
Leukopenia	(8) 57.1%	(6) 42.9%	
Hospital stay (days)	21.95 (±20.94)	25.18 (±25.51)	0.486

Abbreviations: *n*, number; $\bar{x}$, average; SD, Standard Deviation.

**Table 5:** Multivariate analysis of ICU mortality.

Raw Estimation (Unadjusted)						Adjusted Estimation		
Variables	Alive (*n* = 71)	Deceased (*n* = 43)	OR	95% CI	*p*	OR	95% CI	*p*
Age (years)	60.3 (17.66)	66.26 (17.08)	1.022	0.998–1.046	0.070	1.029	1.003–1.055	0.028
Leukocytes	12.58 (8.79)	27.82 (56.83)	1.017	0.997–1.038	0.099	1.024	1.000–1.048	0.048
Creatinine	1.82 (3.26)	1.27 (1.34)	0.911	0.760–1.093	0.317	0.747	0.566–0.985	0.039
Potassium	4.02 (0.66)	4.24 (0.79)	1.575	0.916–2.707	0.100	2.946	1.364–6.361	0.006
Diagnostic accuracy of the associative model								
Cox and Snell R Square				0.170				
Nagelkerke R Square				0.231				
Hosmer–Lemeshow test				0.065				
Sensitivity				76.9%				
Specificity				73.9%				
Validity index				74.6%				
+predictive value				46.5%				
−predictive value				91.5%				
Prevalence				22.8%				
Youden’s index				72.3%				
+likelihood ratio				2.95				
−likelihood ratio				0.30				

Abbreviations: OR: odds ratio; CI: confidence interval; quantitative variables: mean (typical deviation); qualitative variables: absolute frequency (relative frequency); leukocytes (×10^3^/uL); creatinine (mg/dL); potassium (mEq/L).

## Data Availability

Not applicable.
